# The Diverse Roles of 17β-Estradiol in Non-Gonadal Tissues and Its Consequential Impact on Reproduction in Laying and Broiler Breeder Hens

**DOI:** 10.3389/fphys.2022.942790

**Published:** 2022-07-01

**Authors:** Charlene Hanlon, Clara J. Ziezold, Grégoy Y. Bédécarrats

**Affiliations:** Department of Animal Biosciences, University of Guelph, Guelph, ON, Canada

**Keywords:** estradiol (17ß-estradiol), reproduction, egg formation, sexual maturation, laying persistency, medullary bone, yolk deposition

## Abstract

Estradiol-17β (E_2_) has long been studied as the primary estrogen involved in sexual maturation of hens. Due to the oviparous nature of avian species, ovarian production of E_2_ has been indicated as the key steroid responsible for activating the formation of the eggshell and internal egg components in hens. This involves the integration and coordination between ovarian follicular development, liver metabolism and bone physiology to produce the follicle, yolk and albumen, and shell, respectively. However, the ability of E_2_ to be synthesized by non-gonadal tissues such as the skin, heart, muscle, liver, brain, adipose tissue, pancreas, and adrenal glands demonstrates the capability of this hormone to influence a variety of physiological processes. Thus, in this review, we intend to re-establish the role of E_2_ within these tissues and identify direct and indirect integration between the control of reproduction, metabolism, and bone physiology. Specifically, the sources of E_2_ and its activity in these tissues via the estrogen receptors (ERα, ERβ, GPR30) is described. This is followed by an update on the role of E_2_ during sexual differentiation of the embryo and maturation of the hen. We then also consider the implications of the recent discovery of additional E_2_ elevations during an extended laying cycle. Next, the specific roles of E_2_ in yolk formation and skeletal development are outlined. Finally, the consequences of altered E_2_ production in mature hens and the associated disorders are discussed. While these areas of study have been previously independently considered, this comprehensive review intends to highlight the critical roles played by E_2_ to alter and coordinate physiological processes in preparation for the laying cycle.

## Introduction—Estrogens and Their Receptors

Estrogens play a fundamental role in controlling female reproduction, with the ovary acting as the primary source. Interestingly, in avian species, estrogen biosynthesis has also been reported in non-gonadal tissues, such as the brain and adrenal glands (Tanabe et al., 1979; Matsunaga et al., 2001). As the synthesis of estrogens in mammals has additionally been observed in the skin, heart, muscle, liver, adipose tissue, and pancreas (Hemsell et al., 1974; Cui et al., 2013; Barakat et al., 2016), there is a rationale for further investigation in the avian species. Three primary forms of estrogens can be found in circulation: estrone (E_1_), estradiol-17β (E_2_), and estriol (E_3_). In the hen, it has been established that E_1_ is a readily reversible estrogen form (MacRae et al., 1959), acting primarily as a precursor and storage form of the most prominent estrogen, E_2_ (Raud and Hobkirk, 1968). In avian species, E_2_ is primarily produced by small white follicles (SWFs) following their recruitment during sexual maturation of the ovary (Williams and Sharp, 1977; Robinson and Etches, 1986). In fact, E_2_ plays an instrumental role in the transition between growth and reproduction in the laying hen. Contrary to mammals, in which E_2_ is primarily produced by granulosa cells (Hutz, 1989), E_2_ synthesis in birds is localized to the theca externa layer prior to follicular selection into the hierarchy (Porter et al., 1989). In comparison to the influence of E_2_, E_3_ is a weak, minor form of estrogen (Lague et al., 1975), which is 80-fold less potent than E_2_ and acts as a major urinary metabolite rather than playing an active role in controlling reproduction (Mathur et al., 1966).

Three estrogen receptors (ERs) have been characterized, including two intracellular receptors, ER-alpha (ERα, also known as ESR1) and ER-beta (ERβ, also known as ESR2), and one cell surface ER, referred to as GPR30 (also known as GPER-1). E_1_ and E_3_ have shown a preference for ERα and ERβ, respectively, while E_2_ maintains a similar binding capacity for either intracellular receptor (Kuiper et al., 1998). Conversely, much less is known about the activity of GPR30, which was first reported to mediate the response to E_2_ in breast cancer cells despite the lack of ERα and ERβ (Filardo et al., 2000; Thomas et al., 2005). Since then, it has been implicated in numerous pathways regulated by E_2_, including germ cell proliferation (Olde and Leeb-Lundberg, 2009; Ge et al., 2012). In fact, during embryonic development, GPR30 has been implicated in the renewal of chicken primordial germ cells (PGCs) induced by E_2_ (Ge et al., 2012). More recent investigations in mice models have indicated that during follicle-stimulating hormone (FSH)-stimulated aromatase production, E_2_ binds to GPR30 to trigger ERK1/2 phosphorylation, resulting in oocyte maturation *in vivo* (Zhao et al., 2020). However, since the discovery of this G-coupled protein ER in chickens is more recent (Acharya and Veney, 2012; Ge et al., 2012), there is little information on its role both in reproduction and metabolic processes. In the case of the intracellular receptors, differences in expression have been identified between the subtypes. Specifically, ERα was identified within the embryonic brain of Japanese quail and chickens (Camacho-Arroyo et al., 2003; Brunström et al., 2009), as well as the hypothalamus of mature laying hens (Hansen et al., 2003), the ovary of mature hens (Hrabia et al., 2004), shell gland (uterus), kidney (Hansen et al., 2003), liver (Li et al., 2014), and bones (Ohashi et al., 1991; Ohashi and Kusuhara, 1993; Turner et al., 1993). In the case of avian ERβ, expression is less widespread, as it was detected in the embryonic brain (Brunström et al., 2009), ovary (Hrabia et al., 2008), and liver (Li et al., 2014).

While both intracellular receptors are expressed in the avian hypothalamus and pituitary, ERα is the predominant subtype (Griffin et al., 1999), and this receptor is primarily implicated in activating the hypothalamic-pituitary-gonadal (HPG) axis. However, expression of ERβ was identified in the embryonic brain region of quail associated with copulatory behaviour in mature males, suggesting a role for the β-subtype in brain differentiation (Brunström et al., 2009). In the ovary, ERα is the predominant ER (Hrabia et al., 2008). While both intracellular ERs are upregulated during follicular development and the ovulatory process (Drummond and Fuller, 2010), the α-subtype in granulosa cells is predominantly involved in triggering follicular recruitment and the maturation of the remainder of the reproductive tract at the time of activation (Kamiyoshi et al., 1986; Hrabia et al., 2004). ERα is also present within the oviduct of hens to trigger the final maturation of the reproductive tract and coordinate the egg formation processes (Hansen et al., 2003; Imamura et al., 2006). In the case of ERβ, this receptor is predominantly involved with the maturation of pre-ovulatory follicles and the ovulatory process (Emmen et al., 2005).

In the bone and liver, ERα once again plays the predominant role (Imamura et al., 2006; Li et al., 2014). While the expression of both ERs in hepatocytes elevates during sexual maturation (Tan et al., 2020), only ERα has been implicated in mediating the upregulation of yolk proteins (Li et al., 2014). GPR30 has also been detected in the liver (Liu et al., 2018), yet little is known regarding its role. Interestingly, in the presence of growth hormone (GH), a significant elevation in ERβ was reported (Hrabia, 2015), suggesting GH may upregulate ERβ in the liver to participate in nutrient partitioning and utilization during lay. ERα is the only receptor subtype reported to support eggshell formation during bone development in the laying hen. Specifically, since ERα is expressed on the surface of osteoblasts (Ohashi et al., 1991; Ohashi and Kusuhara, 1993; Turner et al., 1993), as the hen ages and transitions toward the end of the production cycle, the receptor density declines, reducing the osteogenic effect of E_2_ (Hansen et al., 2003). However, the recent determination that modern commercial laying hens can persistently lay up to 100 weeks of age with no detrimental impact on bone or eggshell quality (Hanlon et al., 2022) suggests that genetic selection may have impacted the response to E_2_. Thus, further studies are required to determine whether this results from a sustained expression of ERs in these strains.

**FIGURE 1:**
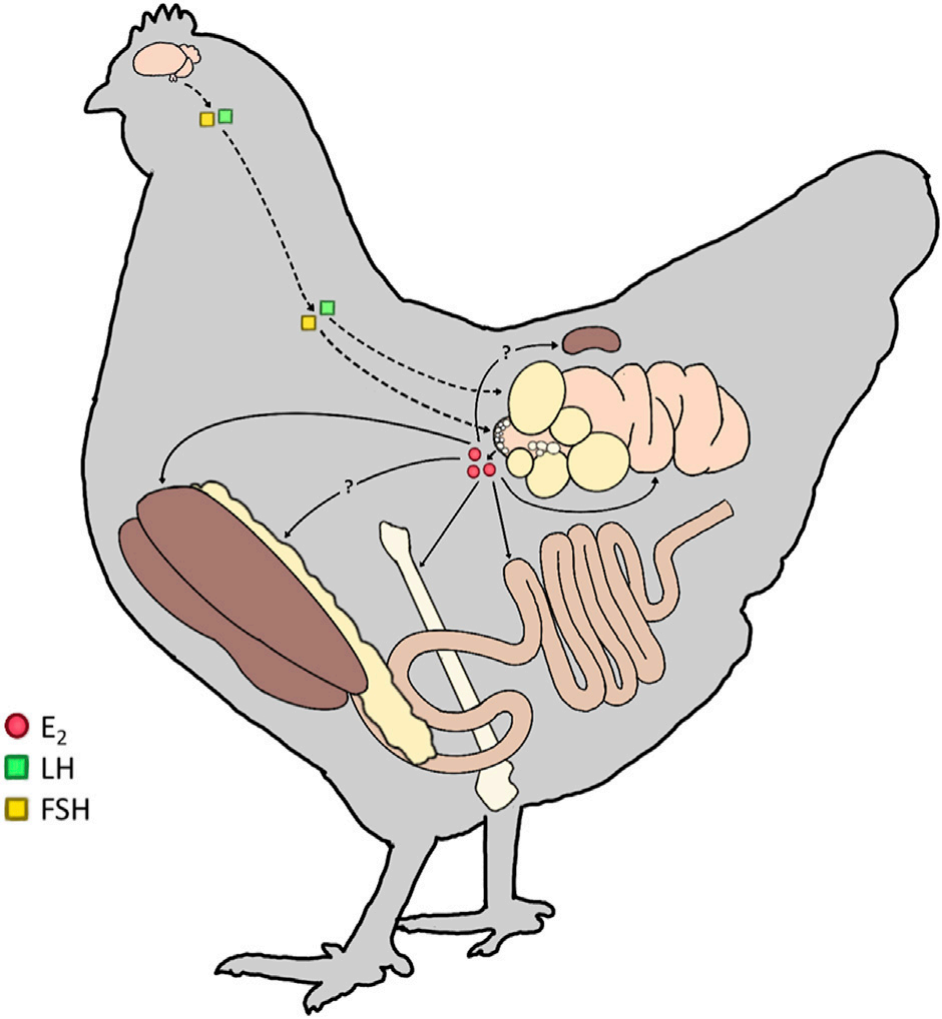
Targets of lowercase (E_2_) in mature hens. In the mature laying hen, the upper portion of the hypothalamic-pituitary-gonadal (HPG) axis is activated, resulting in the production of gonadotropins (Luteinizing Hormone; LH and Follicle-Stimulating Hormone; FSH). FSH and LH then act on the ovary (dashed line) to stimulate follicular development and ovulation, respectively. The small white follicle (SWF) pool synthesizes and releases E_2_ from the ovary (solid line). E_2_ targets estrogen receptors (ERs) in the liver, bone, intestine, and oviduct to participate in all levels of the egg formation process. We hypothesize additional ERs are present in the adipose tissue and kidney of hens to further integrate the physiological processes during sexual maturation and the preparation for lay.

Furthermore, we hypothesize that ER activity likely extends beyond the investigated tissues, as ERs are expressed in adipose tissue in mammals. In avian species, while the follicle-stimulating hormone receptor (FSH-R) has been identified in adipose tissue (Cui et al., 2012), the presence of ERs has yet to be determined. Therefore, it is possible that E_2_, via its receptors, alters adipocyte functions, diverting nutrients and energy toward egg production through the upregulation of ERs by FSH. Thus, for the remainder of this review, the focus is placed on the roles of E_2_ as the primary estrogen during sexual differentiation of the embryo, maturation of the hen, and extended laying cycle. Moreover, this review will emphasize the roles of E_2_ in yolk formation, skeletal development, and metabolism, which all impact egg production and quality (Figure 1). Lastly, the consequences and disorders associated with varying E_2_ concentrations are discussed to provide a comprehensive review and demonstrate that beyond the reproductive tract, E_2_ is key to altering metabolic processes during the life cycle of a laying hen.

## Estradiol and Reproduction

### Embryonic Sexual Differentiation

Aside from the gonadal sources of E_2_, studies have reported the presence of maternally derived estrogens in the yolk (Schwabl, 1993; Paitz et al., 2020), potentially implicating estrogen in all stages of embryonic development. A study in quail determined that the E_2_ concentration present in yolk is comparable to that of the maternal circulating concentrations (Adkins-Regan et al., 1995). In fact, chicken embryos have concentrations as high as 0.2 ng/g of body weight (Elf and Fivizzani, 2002; Hartmann et al., 2003; Wang et al., 2010), and in other avian species, this concentration is as high as 12 ng/g (Merrill et al., 2019). Since ERα expression has been reported as early as embryonic day 3.5 (E3.5; Andrews et al., 1997; Smith et al., 1997), prior to the production of aromatase (Smith et al., 1997) and E_2_ by the gonads (Woods and Erton, 1978), these maternally derived sources of estrogen may be the primary source acting on the ERs during this period. Additional sources of E_2_ are produced in the interstitial cells, irrespective of sex, between E3.5–5.5 (Woods and Erton, 1978). However, the role of estrogen in this early developmental period remains to be determined, as it was recently established that E_2_ is metabolized to estrone early *in ovo*, then conjugated to sulfates and glucuronides by E5 (Paitz et al., 2020). In female quail treated with estradiol benzoate, female offspring developed right oviducts, resulting in an atypical symmetrical reproductive tract morphology (Adkins-Regan et al., 1995). It has been speculated that the early metabolism of maternally derived E_2_ observed by Paitz et al. (2020) suppresses the metabolic capacity for Mullerian duct development on the right side (Zimmerman et al., 2012).

**FIGURE 2:**
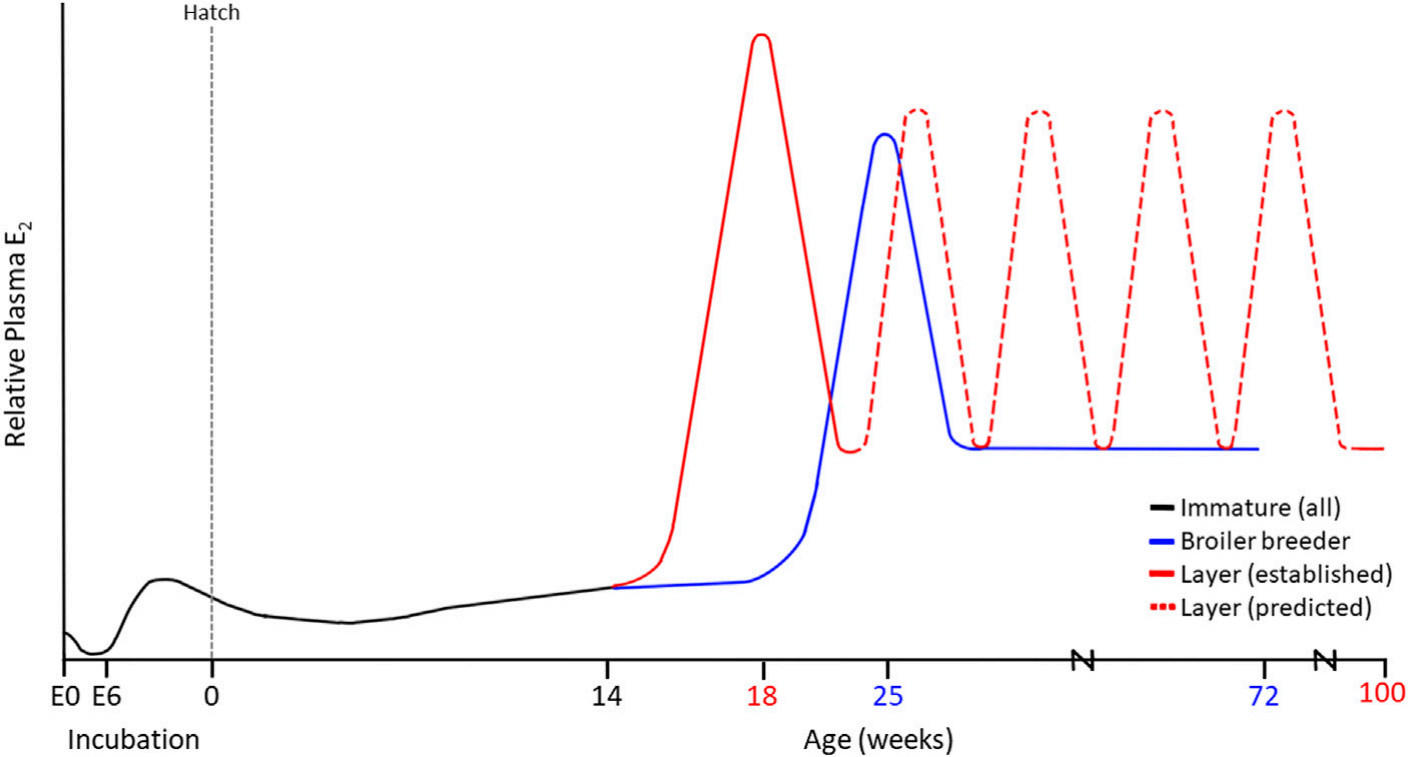
Established and Proposed Estradiol (E_2_) Profiles in Modern Commercial Laying Hens (100 weeks) and Broiler Breeder Hens (72 weeks). Maternal sources of E_2_ are present in the embryo at day 0 (E0), prior to conjugation during early incubation. At E6.5, the production of aromatase stimulates the ZW left ovarian cortex to produce E_2_, driving sexual differentiation. These levels decrease around the time of hatch as the chick ovary remains in a quiescent state. Maturation varies among layers and broiler breeders, yet both demonstrate an initial peak in E_2_. In the case of the layer, we propose that up to 5 additional recurrent E_2_ elevations occur throughout lay, contributing to the persistency in the cycle that is not observed in broiler breeder hens.

Estrogens have long been implicated in the feminization of the reproductive axis during sexual differentiation, with early exposure to estrogen critical for the normal development of the ovary (Elbrecht and Smith, 1992). Thus, female embryos of turkeys, quail, and chickens have higher circulating E_2_ concentrations compared to their male counterparts (Woods and Brazzill, 1981; Schumacher et al., 1988; Abdelnabi et al., 2001; Ottinger et al., 2001; Sechman et al., 2011; Rosati et al., 2021). Recent evidence demonstrates that *JUN* is a critical regulator of sexual differentiation, regulating *DMRT1*, *SOX9*, and *FOXL2*, the genes previously hypothesized to be the determinants of differentiation in avian species. In fact, overexpression of *JUN* results in feminization of ZZ embryos and *JUN* knockout of ZW embryos results in masculinization (Zhang et al., 2021). Upregulation of *JUN* results in the inhibition of Smad2 and the simultaneous production of E_2_ within the gonad (Zhang et al., 2021). These gonadal sources of E_2_ were detected following the activation of P450_17α_ (CYP17A1) as early as 5–6 days into embryonic development in both the right and left ovaries, with the initiation of cytochrome P450_aromatase_ (CYP19A1) and 3-beta-hydroxysteroid dehydrogenase (3β-HSD) by E6.5 (Yoshida et al., 1996; Andrews et al., 1997; Nakabayashi et al., 1998; Nomura et al., 1999; Nishikimi et al., 2000; Figure 2). In fact, the W chromosome has been linked to the early aromatase activation in the female embryo, leading to ovarian development during the first half of the incubation period (Ottinger, 1989; Kagami and Hanada, 1997). It has even been suggested that the presence of CYP19A1 is not only necessary but sufficient to initiate of female sexual differentiation in the embryo (Jin et al., 2020). This elevation in E_2_ is key to activating R-spondin-1 (*RSPO1*) expression during early embryonic development, thereby regulating the WNT/β-catenin signalling pathway responsible for controlling cell fate and stem cell pluripotency (Smith et al., 2008).

While the elevated synthesis of E_2_ will suppress anti-Mullerian hormone receptor II (AMHRII) to protect the Mullerian duct from apoptosis (Cutting et al., 2014), this protection only occurs in the left ovarian cortex of avian species. This results in an asymmetrical reproductive tract development, as the expression of ERα in the right ovarian cortex is suppressed under the influence of the pituitary homeobox 2 (PITX2) (Nakabayashi et al., 1998). E_2_ cannot exert its effects without its receptor present in the cortex, causing the right ovarian cortex to begin regressing as early as E6-6.5 (Ishimaru et al., 2008). Conversely, ERα is expressed in the medulla and germinal epithelium of the left ovary as early as E5.5 (Mizuno et al., 1996; Yoshida et al., 1996; Nakabayashi et al., 1998; Ishimaru et al., 2008). Interestingly, as ERα is temporarily expressed in male embryos between E7-10 (Nakabayashi et al., 1998), embryos can undergo sex reversal following exposure to supraphysiological doses of exogenous E_2_. This results in the development of ovotestes at hatch, despite the presence of ZZ chromosomes (Scheib, 1983). However, regardless of E_2_ administration, differentiation into testis was shown to eventually occur during sexual maturation (Etches and Kagami, 1997). Interestingly, supraphysiological doses of E_2_ are also detrimental to the development of the female reproductive tract, resulting in an intact right ovary and abnormal formation of the left Mullerian duct (Rissman et al., 1984). Therefore, an E_2_ threshold likely exists during embryogenesis for normal gonadal development. This may include maternal sources accumulated within the yolk, which may in turn impact hatch rate and future reproductive capacity of offspring.

### Estradiol Production and Ovarian Follicular Development

In avian species, the reproductive axis is under the control of a dual stimulatory and inhibitory system, mediated through the hypothalamic neuropeptides, gonadotropin-releasing hormone-I (GnRH-I) and gonadotropin-inhibitory hormone (GnIH) (for review: Bédécarrats, 2015; Bédécarrats et al., 2016; Hanlon et al., 2020). Briefly, long-day breeders, such as the domestic chicken, respond to increasing day lengths, also referred to as photostimulation, by downregulating the expression of GnIH and activating the release of GnRH-I. As GnIH directly suppresses GnRH (Bentley et al., 2003, 2008) and gonadotropins (Tsutsui et al., 2000; Ciccone et al., 2004; Ikemoto and Park, 2005; Ubuka et al., 2006), the downregulation of GnIH allows for the full activation of the reproductive axis (Ikemoto and Park, 2005; Maddineni et al., 2008). In turn, the anterior pituitary gland releases gonadotropins, luteinizing hormone (LH) and FSH, which enter the systemic circulation.

Prior to sexual maturation, LH stimulates a gradual increase in ovarian E_2_ production from thousands of SWFs (Williams and Sharp, 1977; Robinson and Etches, 1986; Sechman et al., 2000; Figure 2). This E_2_ stimulates the final developmental stage of the oviduct by mediating epithelial cell differentiation into tubular gland cells, goblet cells, and ciliated cells (Kohler et al., 1969), thus allowing for albumen synthesis and deposition during egg formation (Palmiter and Wrenn, 1971). Activation of the reproductive axis is concurrent with follicular development via cyclic recruitment, resulting in a mature, functional ovary. Cyclic recruitment in the avian ovary is characterized by a distinct arrangement of prehierarchal and hierarchical (F1-F6) follicles (Johnson, 1993), in which the largest follicle (F1) is ovulated daily (Johnson et al., 1996). FSH supports the maintenance of prehierarchal follicles via proliferative signals (Johnson, 2003) that reduce the susceptibility of developing granulosa cells to apoptosis (Johnson et al., 1996, 1999), thus preventing follicular atresia. Furthermore, LH and FSH are both capable of initiating receptor-mediated cAMP production through their respective receptors, resulting in steroidogenesis (Porter et al., 1989; Kowalski et al., 1991). However, compared to FSH, LH has a more potent effect on steroid hormone secretion (Sharp et al., 1979; Robinson et al., 1988). LH stimulates progesterone (P_4_), testosterone (T), and E_2_ production from theca cells, with higher steroid concentrations produced by less mature follicles. However, the primary product of the theca interna layer is T, which is subsequently converted to E_2_ by the theca externa cells (Porter et al., 1989). In the developing granulosa cells, FSH induces the necessary steroidogenic competence (Tilly et al., 1991) for P_4_ synthesis and LH receptor expression in small yellow follicles and intermediate preovulatory follicles (Calvo and Bahr, 1983; Tilly et al., 1991; Johnson and Bridgham, 2001). As preovulatory follicles mature, granulosa cells lose FSH sensitivity and become exclusively LH-responsive. For instance, in the three largest follicles (F1, F2 and F3), LH increases granulosa cell cAMP production, whereas FSH has no effect (Calvo et al., 1981). In the mature preovulatory follicle (F1), LH stimulates the secretion of high concentrations of P_4_ (Yang et al., 1997). This P_4_ release exerts positive feedback on GnRH-I, which triggers the preovulatory LH surge (Johnson and van Tienhoven, 1980), inducing ovulation 4–7 h later (Furr et al., 1973). Interestingly, under the influence of the anti-estrogen tamoxifen, egg-laying ceased despite an elevation in E_2_ production by SWFs. This occurred concomitantly with declining P_4_ concentrations, leading to the inhibition of ovulation (Rzasa et al., 2009).

While E_2_ exerts negative and positive feedback on GnRH via kisspeptin neurons in mammals, the absence of kisspeptin in birds may explain the predominantly inhibitory feedback of E_2_ on gonadotropins. Removing the primary source of E_2_ through ovariectomy induces a substantial rise in circulating LH concentrations in pullets (Knight et al., 1981) and increases pituitary LHβ subunit mRNA levels in mature hens (Terada et al., 1997). Furthermore, E_2_ replacement following ovariectomy reduces plasma LH in adult hens (Wilson and Sharp, 1976; Terada et al., 1997) and downregulates pituitary LHβ subunit mRNA levels (Terada et al., 1997). Similarly, E_2_ injection suppresses circulating levels of LH and FSH in photostimulated birds; however, this response is age-dependent (Dunn et al., 2003). E_2_ may mediate the acquisition of the neuroendocrine response to photostimulation, as E_2_ treatment combined with exposure to long daylengths increases plasma gonadotropin concentrations in pullets (Dunn et al., 2003). Likewise, treatment with E_2_ or a combination of E_2_ and P_4_ reduces GnIH-R mRNA abundance in the pituitary of pullets, likely reducing the response to GnIH treatment in adult hens (Maddineni et al., 2008). Interestingly, priming with E_2_ is also required to develop the LH positive feedback response to P_4_ necessary for ovulation (Wilson and Sharp, 1976), through E_2_-induced P_4_ receptor (PR) expression in the pituitary gland (Gasc and Baulieu, 1988). At the level of the hypothalamus, continuous E_2_ treatment was shown to upregulate PR in immature females, with receptor levels returning to baseline following E_2_ withdrawal (Gasc and Baulieu, 1988). This suggests that E_2_ plays a stimulatory role in priming the HPG axis for maturation. However, daily tamoxifen injections in mature layers also resulted in elevated ovarian E_2_ production (Mao et al., 2018), which indicates that E_2_ feedback mechanisms in birds may be more comparable to mammals than hypothesized.

### Estradiol and Oviduct Formation

During the development of the chick, implants containing estrogen diethylstilbestrol (DES) triggered cellular proliferation and prevented apoptosis in the developing oviduct (Monroe et al., 2000, 2002). Doses of up 12,000 to 25,000 IU of estradiol benzoate administered to chicks resulted in a 10-fold increase in diameter (Wolff, 1936), and doses of α-estradiol dipropionate at 4 mg per 100-g of body weight led to an 80-fold increase in oviduct weight (Munro and Kosin, 1943). Although the magnum remains an undifferentiated epithelial tube during the immature state, daily injections of estradiol benzoate beginning at 4 days of age can result in an 8-fold increase within 3-days (Palmiter and Wrenn, 1971), indicating that the chick oviduct is already prepared to respond to E_2_ stimulation in the days following hatch. This exogenous E_2_ during chick growth can also trigger an elevated expression of ovalbumin (Kohler et al., 1969; Palmiter and Wrenn, 1971). These levels will fall quickly in the absence of E_2_ stimulation (Schimke et al., 1975). As the hen enters maturity, E_2_ mediates the differentiation of tubular gland cells in the magnum, which can be inhibited by the simultaneous administration of P_4_ (Oka and Schimke, 1969) through the reduction of cytoplasmic, nuclear, and total ERα (Hsueh et al., 1976; Kraus and Katzenellenbogen, 1993).

The shell gland segment of the oviduct contains a large amount of calbindin 28 K (Corradino et al., 1968) and receptors for cholecalciferol (1,25(OH)_2_D_3_) (Coty, 1980; Haussler, 1986). The binding of cholecalciferol to its receptor triggers an upregulation of the calbindin gene (Theofan et al., 1986; Clemens et al., 1988; Mayel-Afshar et al., 1988). In turn, calbindin protein is responsible for binding calcium and, in the case of the shell gland, depositing this calcium as eggshell in the form of calcium carbonate (Bradfield, 1951; Corradino et al., 1968). Calbindin concentrations are also stimulated by E_2_ (Navickis et al., 1979; Nys et al., 1986, 1989), as calbindin is colocalized with ER within the glandular epithelial cells (Jande et al., 1981; Isola, 1990). Thus, levels of this protein increase at the time of sexual maturation (Nys et al., 1989). However, during the formation of the first egg, the expression of calbindin is highly dependent on the transfer of calcium to the shell gland rather than E_2_ concentrations (Nys et al., 1989; Striem and Bar, 1991).

Oviductal prolapse is the cause of up to 20% of mortality in laying hens (Shemesh et al., 1984). Evidence has shown increased incidence with declining E_2_ levels in the blood (Shemesh et al., 1982). Interestingly, not only has E_2_ treatment been shown to support recovery, but spontaneous recovery has also been associated with sudden elevations in E_2_ (Shemesh et al., 1984). Recovered birds had higher E_2_ in the theca layer of the follicles compared to healthy or prolapsed counterparts (Shemesh et al., 1984). Thus, it has been proposed that prolapse results from systemic E_2_ deficiency.

## Estradiol and Metabolism - Yolk Formation

### Activation of the Liver

The liver undergoes two metabolic states in hens: pullet growth and active laying (Gruber et al., 1976). Prior to the elevation of E_2_ during sexual maturation, the liver primarily contributes to pullet growth. In fact, the liver is responsible for ∼90% of fatty acid synthesis (Leveille et al., 1968; O’Hea and Leveille, 1968; Wang et al., 2014), producing a generic VLDL for the transport of dietary lipids in the blood (Chapman et al., 1977; Walzem, 1996; Walzem et al., 1999). These VLDLs are considerably large (∼70 nm diameter) due to their association with 6 identified apolipoproteins, including apoA-1, apoB, and apoC (Walzem, 1996). Thus, these lipids will only be stored in adipose or utilized by peripheral tissues (Walzem, 1996), as they are rapidly processed and energy inefficient, with only ∼40% of the substrate used (Griffin et al., 1982). At this time, lipid transport is quite similar to that in mammalian species, with the exception of dietary fat being transported as portomicrons through the portal vein in immature birds (Bensadoun and Rothfeld, 1972) rather than through the lymphatic system in mammals. Upon the initiation of egg-laying, traditional VLDLs are too large to undergo deposition into the growing follicle (Walzem et al., 1999), while intermediate-density lipoproteins (IDLs) and low-density lipoproteins (LDLs) are smaller particles but provide insufficient energy (Griffin et al., 1982). Thus, a modified lipoprotein is required to meet the demands of yolk deposition.

**FIGURE 3:**
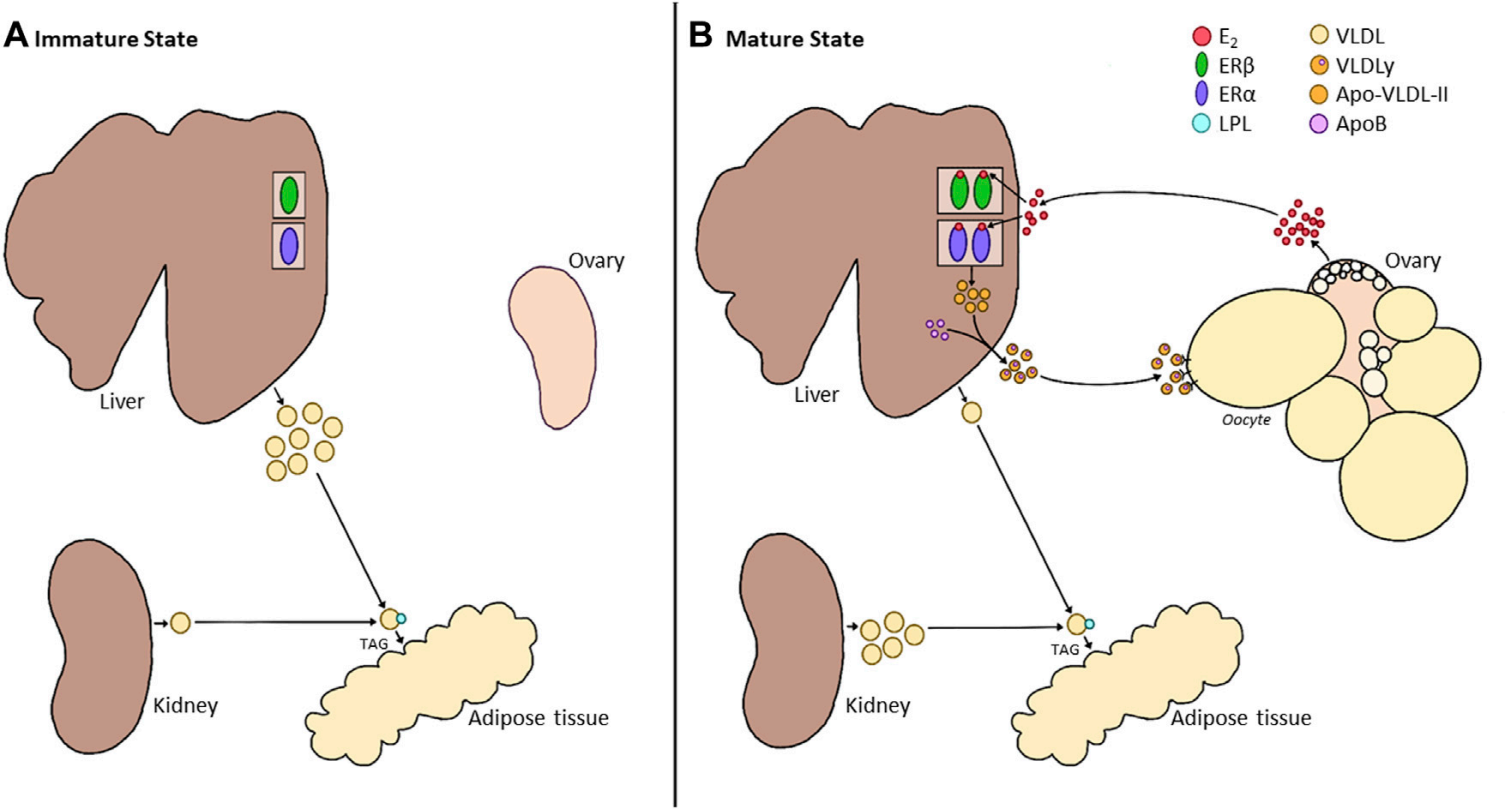
Alterations in Liver Metabolism between the Immature and Mature States of Hens. **(A)**. During the immature state (pullet), the ovary of the bird remains in a quiescent state. While the liver already expresses estrogen receptors (ERα and ERβ), the absence of E_2_ results in the production of a generic VLDL. This VLDL is broken down into triglycerides (TAGs) by lipoprotein lipase (LPL) in the circulation and deposited into tissues such as the adipose. During this time, the kidney produces a small amount of generic VLDL and minor production of E_2_ by the immature ovary. **(B)**. Once the ovary begins to produce E_2_, this hormone binds to its receptors in the liver. While little is known about the role of ERβ in the liver, binding to ERα results in the production of a smaller, more energy-efficient VLDLy, made up of ApoVLDL-II and ApoB in a 23:1 ratio. VLDLy is resistant to LPL, aiding in the deposition of this yolk protein in the rapidly growing follicle. While a small portion of generic VLDL can be produced within the liver, the primary source for peripheral tissues will now be provided by the kidney. However, the role of E_2_ in the switch of kidney function is still undetermined.

At the time of sexual maturation, ERα present in the liver binds E_2_ to elicit the expression of estrogen-dependent genes driving yolk production and enhancing the stability of genes associated with egg white proteins (Bergink and Wallace, 1974; Kirchgessner et al., 1987; Flouriot et al., 1996). Therefore, under the influence of E_2_, the liver produces VTG-II (Deeley et al., 1977; Evans et al., 1987), a glycophospholipoprotein that provides the oocyte with glucose, phosphorous, and fat while binding metal ions such as calcium, zinc, and iron (Montorzi et al., 1995). E_2_ also stimulates the liver to produce ApoVLDL-II (Chapman et al., 1977; Deeley et al., 1977; Wiskocil et al., 1980; Codina-Salada et al., 1983; Evans et al., 1987; Ratnasabapathy, 1995; Flouriot et al., 1996; Ratnasabapathy et al., 1997; Walzem et al., 1999), an apolipoprotein that inhibits lipoprotein lipase (LPL) to prevent susceptibility to the breakdown of the triglycerides (TAGs) during transport to the oocyte (Griffin et al., 1982; Schneider et al., 1990). Laying hens also have lower LPL activity in the adipose and heart and adipose tissue than their immature counterparts (Husbands, 1972), supporting the redirection of VLDLs to the ovary (Perry and Gilbert, 1979). This Apo-VLDL-II is packaged in a 23:1 ratio with ApoB, combining to form a yolk-targeted TAG-rich VLDL (VLDLy) (Walzem et al., 1999). Due to its association with only two apolipoproteins, the size of VLDLy is reduced by ∼50% (30 nm) without sacrificing energy, TAG, or phospholipid content (Griffin et al., 1982; Griffin and Perry, 1985). This allows for easier transport to the ovary, where they can be incorporated into the oocyte via receptor-mediated endocytosis and pass through the granulosa layer (Perry and Gilbert, 1979; Krumins and Roth, 1981; Griffin et al., 1982). Therefore, VLDLy is an ideal candidate for yolk deposition due to the high requirement of energy to be packaged into the follicle for embryo development (Kudzma et al., 1979; Griffin, 1981; Dashti et al., 1983; Lin and McCormick, 1986; Chan et al., 1990; Schneider et al., 1990; Speake et al., 1998; Walzem et al., 1999). These VLDLy particles make up ∼30% of the total weight of each yolk, with hens producing up to 5 g of lipids for the daily yolk accumulation (Griffin et al., 1982). Therefore, to keep up with the demand, VLDLy synthesis is significantly upregulated by E_2_ compared to the previous production of generic VLDL (Walzem et al., 1999). Furthermore, E_2_ has been implicated in the upregulation of lipolytic activities and facilitation of yolk transport during the rapid growth phase of the preovulatory follicles (Brady et al., 1976; Deeley et al., 1977; Wiskocil et al., 1980; Cochrane and Deeley, 1988; Speake et al., 1998; Walzem et al., 1999; Nikolay et al., 2013). Finally, E_2_ also stimulates the production of albumen, which is transported to the oviduct during egg formation (Deeley et al., 1975; Cochrane and Deeley, 1988; Evans et al., 1988; Li et al., 2014; Ratna et al., 2016), and is thus critical to the successful formation all internal egg components (Figure 3).

Studies have also investigated the impact of exogenous E_2_ sources prior to the activation of the HPG axis, and a similar response was observed (Balnave, 1971; Muramatsu et al., 1992; Elnager and Abd-Elhady, 2009), suggesting that ERs are expressed and responsive during pullet growth in preparation for maturation. While it has been established that the populations of both nuclear ER subtypes increase during sexual maturation (Tan et al., 2020), the precise age at which ERs are expressed has not been established. Nonetheless, VLDLy production, along with the associated yolk precursors, apo-VLDL-II and VTG-II, were found to elevate prior to reproductive maturity in response to exogenous E_2_ (Muramatsu et al., 1992). This was further associated with increased liver weight and plasma lipid concentrations (Balnave, 1971; Elnager and Abd-Elhady, 2009). In fact, the TAG serum content elevations are associated with a greater number of lipid droplets in the liver of E_2_-treated hens than the control (Ren et al., 2021), demonstrating the direct impact of E_2_ on liver physiology. Interestingly, while E_2_ is required to activate yolk precursors and E_2_ concentrations decline in aging hens, a recent study showed that ApoVLDL-II and VTG-II mRNA elevate with age (Cui et al., 2020). A similar result was observed in Li et al. (2020), in which Apo-VLDL-II and ApoB increased over time. This is in line with the hypothesis that the production of yolk precursors may be linked to the recurrent elevations in E_2_ observed in commercial strains (Hanlon et al., 2021), suggesting that E_2_ may play a larger role in maintaining the egg-laying cycle than previously hypothesized.

### Liver Response

More recently, additional estrogen-responsive genes expressed in the liver have been under investigation (Zhang et al., 2020; Ren et al., 2021). Upon exogenous E_2_ administration in mature laying hens, a 23-fold elevation in peroxisome-proliferative receptor γ (PPARγ) expression was observed (Lee et al., 2010). This corresponded to increased fatty acids, TAGs, and hepatic lipids (Sato et al., 2009; Lee et al., 2010). Higher hepatic expression of PPARγ has previously been associated with a higher production rate (Chen et al., 2010) and a decline occurs with age (Cui et al., 2020). However, since the expression patterns of ERα increase in aging hens while ERβ does not change (Cui et al., 2020), circulating levels of estrogens are likely the most critical factor.

Apela, which is speculated to be involved in the hepatic lipid metabolism of hens, is activated by E_2_ in the liver during embryogenesis via ERα. This gene can be induced with exogenous E_2_, as demonstrated by the elevated expression at the peak of lay (Tan et al., 2020). Estrogen is also reported to bind to ERα to downregulate *gga-miR-221-5p*, increasing the expression of *ELOVL6* and *SQLE*, responsible for stimulating lipid synthesis, consistent with the role outlined above (Zhang et al., 2020). E_2_ binding to ERα also directly targets *miR-144* (Vivacqua et al., 2015), which is known to regulate lipid metabolism through the suppression of *PPARγ-coactivator-1β* (*PGC-1β*; Ren et al., 2021). Furthermore, *PGC-1α* is also influenced by ERα and ERβ (Jiang et al., 2021). However, the action of E_2_ on *PGC-1α* remains unclear. Further investigation may reveal a mechanism through which E_2_ can directly control lipogenic genes in the liver to influence adipose accumulation and deposition. The involvement of ERα is particularly unique and important to note, as ERβ has been the primary receptor subtype suggested to play a role in the metabolic control of E_2_ (Li et al., 2015, 2018). Instead, ERα may be an underappreciated potential target for treating metabolic disorders. However, that does not imply that ERβ is not involved. In fact, *cathepsin E-A like* gene, suggested to play a role in the cleavage and processing of VTGs and ApoB (Wang et al., 2022), acts through the β-receptor (Zhang et al., 2018), demonstrating that both receptors will play a critical role in the yolk formation process.

### Activation of the Kidney

Comparable to the liver, the activity of the kidney also depends on the maturity status of the hen. Initially, it was believed that E_2_ did not play any role in the synthesis of ApoB in the kidney or intestine (Kirchgessner et al., 1987; Lazier et al., 1994), while it was shown to directly control the production of ApoVLDL-II in the embryonic kidney (Lazier et al., 1994). However, the hen’s kidney produces generic VLDL and HDL (Bensadoun and Rothfeld, 1972; Tarugi et al., 1998), with the generic VLDL secreted by cells of the proximal tubules. Thus, generic VLDL from the kidney during the laying cycle was proposed to provide the necessary energy to tissues evaded by VLDLy (Walzem et al., 1999). While a direct or an indirect role for E_2_ in the production of generic VLDL is inconclusive, low-density lipoprotein receptor-related protein-2 (LRP2) is primed by E_2_ in the kidney during the maturation period (Plieschnig et al., 2012). This endocytic receptor is proposed to be critical in improving the uptake of apolipoproteins to generate and supply generic VLDL to extraoocytic tissues. This is further supported by the elevation of this receptor in the mature hen compared to a mature rooster (Plieschnig et al., 2012). Additionally, ApoB content is higher in the kidney and intestine of laying hens compared to broilers, while no differences are present between breeds in the liver (Zhang et al., 2007). Therefore, it is highly likely that E_2_ is involved in the activity of the kidney following the maturation phase.

### Fatty Liver Hemorrhagic Syndrome

Fatty liver hemorrhagic syndrome (FLHS) is a metabolic condition typically associated with high-producing laying hens (Trott et al., 2014). This disorder was first described by Couch (1956), with the terminology coined by Wolford and Polin (1972) to distinguish FLHS from non-hemorrhagic fatty liver syndrome (FLS). A high rate of lay has long been attributed to FLS or hepatic steatosis in birds, characterized by the rapid accumulation of lipoproteins in the liver. During this time, the rate of hepatic lipogenesis exceeds that of fat mobilization. FLS is typically observed in modern commercial laying hens since the production cycle requires an exceedingly high rate of lipogenesis, thus overwhelming hepatocytes (Polin and Wolford, 1973; Harms et al., 1982; Hansen and Walzem, 1993; Hermier, 1997; Scheele, 1997). The progression of this disorder eventually leads to a decline in egg production rate (Walzem et al., 1993; Shini and Bryden, 2009; Lee et al., 2010; Jiang et al., 2013; Trott et al., 2014).

While pullets and low-producing hens also have some degree of fat infiltration in the liver (Shini et al., 2020), there are fewer and smaller fenestrae in the sinusoidal endothelium of the liver compared to the mammalian species (Fraser et al., 1986; Braet and Wisse, 2002). It has been hypothesized that the size of fenestrated cells helps avoid dietary fat from overwhelming hepatocytes and prevents atherosclerosis (Fraser et al., 1986). Interestingly, E_2_ has also been shown to inhibit coronary atherosclerosis (Hanke et al., 1996; Beaufrère, 2013; Petzinger and Bauer, 2013). Since large particles are blocked from passage, they remain trapped in sinusoids around the central vein, resulting in FLS. This can eventually progress to FLHS with the continued accumulation of fat in the liver, overwhelming the hepatic system (Shini and Bryden, 2009).

Typically, severe cases of FLHS results in the sudden death of older laying hens during their post-peak production (Butler, 1976; Shini et al., 2012), with up to 42% of mortality observed in commercial flocks and up to 74% of caged hen mortality, making this the number one cause of non-infectious laying hen mortality (Shini et al., 2006, 2019; Shini and Bryden, 2009; Mete et al., 2013; Trott et al., 2014). While this metabolic disorder was previously believed to be a result of heat stress (Ringer and Sheppard, 1963; Wolford, 1971; Schexnailder and Griffith, 1973; Pearson and Butler, 1978a; Rozenboim et al., 2016), caged environments (Squires and Leeson, 1988), and high lipid diets (Couch, 1956; Jaussi et al., 1962; Bragg et al., 1973; Haghighi-Rad and Polin, 1982), further evidence has suggested the poorly defined pathogenesis of this disorder is much more complex. Excessive consumption of high energy, low protein (HELP) diets has been clearly established to induce FLHS (Hermier et al., 1994; Lee et al., 2010). While these diets are incredibly effective as a model for this condition, it is well-recognized that the dietary onset of FLHS is also correlated with elevations in E_2_ concentrations (Butler, 1976; Polin and Wolford, 1977; Pearson and Butler, 1978b). Even increasing the metabolizable energy in the diet of broiler breeder hens was shown to correspond to higher E_2_ concentrations (Hadinia et al., 2020). This resulted in earlier lay, higher GnRH mRNA levels and, as anticipated, higher relative lipid content in the carcass (Hadinia et al., 2020). Therefore, the administration of exogenous E_2_ is also highly effective at inducing FLHS independently (Butler, 1976; Polin and Wolford, 1977; Pearson and Butler, 1978b).

Hormonal control of FLHS was first proposed by Butler (1976) since this metabolic disorder was solely accredited to hens presently in lay. Thus, the role of E_2_ was investigated, and studies confirmed that exogenous administration stimulates lipogenesis in immature birds, resulting in hepatic steatosis (Lorenz, 1954; Balnave, 1968, 1971; Yu and Marquardt, 1973; Balnave and Pearce, 1974; Harms et al., 1977; Tarlow et al., 1977; Pearson and Butler, 1978b; Kudzma et al., 1979; Dashti et al., 1983; Klimis-Tavantzis et al., 1983). However, this condition was more consistently induced during exogenous E_2_ administration in mature layers (Harms et al., 1977; Polin and Wolford, 1977; Pearson and Butler, 1978b; Stake et al., 1981).

Hepatic steatosis has been shown to have detrimental effects on E_2_ metabolism in both humans and birds (Adlercreutz, 1970; Haghighi-Rad and Polin, 1981). Despite elevated E_2_ levels during sudden FLHS outbreaks, P_4_ remains unchanged, and production levels decline as liver fat and FLHS scores increase (Haghighi-Rad and Polin, 1981). This is followed by the rapid regression of the ovary and oviduct (Stake et al., 1981). Reduced LPL activity associated with sexual maturation and the switch in liver metabolism at this time results in reduced clearance of fat in the adipose tissue and increased deposition in the liver (Hasegawa et al., 1980). Thus, the liver fat content of laying hens, typically ∼40% of dry weight, can reach up to 70% (Squires and Leeson, 1988). Additionally, E_2_ will trigger hypercholesterolemia and hypertriglyceridemia (Shini et al., 2020) as the clearance rate of E_2_ falls short of its production by the ovary (Hawkins et al., 1969; Johnson and van Tienhoven, 1981; Tsang and Grunder, 1984), resulting in further impaired liver function (Shini et al., 2020). This suggests E_2_ concentrations may become detrimental above a threshold level. Interestingly, white strains are more tolerant to higher and persistent E_2_ concentrations (Stake et al., 1981).

While it has been proposed that a combination of E_2_ and a positive energy balance via high-energy diets is required to induce FLHS (Harms et al., 1977; Polin and Wolford, 1977; Pearson and Butler, 1978b; Stake et al., 1981; Haghighi-Rad and Polin, 1982), Walzem et al. (1994) hypothesized that providing excessive energy to these hens results in decreased E_2_. This reduction would effectively shut down ApoVLDL-II synthesis and secretion, returning the VLDL particles to their larger, generic size (Walzem et al., 1994). However, further investigation demonstrated that E_2_-treated hens with low feed intake have the greatest incidence of FLHS (Stake et al., 1981), concluding that this disorder is primarily under the control of E_2_, and the remainder of the associated factors may be co-morbidities. Additionally, the administration of tamoxifen, an E_2_ inhibitor, reduced the severity of hepatic hemorrhages (Stake et al., 1981). With the updated E_2_ profiles of recurrent elevations linked to persistency, we hypothesize this will impact liver lipogenesis, and it is critical to determine if these elevated concentrations result in the induction of FLHS.

## Estrogens and Bone Formation

### Hormonal Regulation of Calcium Homeostasis

There are two phases of calcium homeostasis regulation in avian species. The first corresponds to structural bone growth occurring throughout the development of the skeletal frame. The second corresponds to the accumulation of medullary bone, which serves as an available pool of calcium for successful egg production. Thus, at the time of sexual maturation, calcium homeostasis shifts from bone growth to calcium storage (Dacke et al., 1993). Regardless of the phase, plasma calcium levels are controlled by the release of parathyroid hormone (PTH), calcitonin (CT), and 1,25-dihydroxycholicaciferol (1,25(OH)_2_D_3_), acting similarly to most mammalian species. However, in the case of chickens, once the hen enters sexual maturity, E_2_ has a major impact on calcium metabolism (Etches, 1987; Newman and Leeson, 1997; Deluca, 2004; Bar, 2008, 2009).

In growing pullets, the majority of calcium is stored as hydroxyapatite (Ca_10_(PO_4_)_6_(OH)_2_), requiring a tight regulation between calcium and phosphorus and adequate dietary inclusion of both minerals for successful bone development (Hurwitz and Bar, 1965; Whitehead and Fleming, 2000). While phosphorus is required to form several egg components, including yolk and albumen (Keshavarz, 1998), requirements remain constant during the laying cycle. Since calcium destined for the eggshell is deposited as calcium carbonate (CaCO_3_), phosphorous is not necessary during this process. Thus, the breakdown of medullary bone results in phosphorous being primarily excreted (Kebreab et al., 2009).

Under natural light, 7-dehydrocholesterol is a precursor synthesized in the liver and transported to the skin to interact with UV light and synthesize cholecalciferol, also known as Vitamin D_3_ (Klassing, 1998b). This form of Vitamin D is the most efficiently metabolized in chickens (Valinietse and Bauman, 1981), thus meeting the demands of the birds without requiring supplementation. However, artificial lighting programs in commercial settings lack UV light, resulting in the inability of birds to synthesize adequate Vitamin D levels (Sharp, 1996). Thus, dietary supplementation with exogenous sources is required, forming 25(OH)D_3_ in the liver (Lund and Deluca, 1966). This form undergoes further hydroxylation in the kidney by the rate-limiting enzyme, 1α-hydroxylase, to produce 1,25(OH)_2_D_3_ (Lund and Deluca, 1966; Gray et al., 1972; DeLuca, 1974, 1976; Haussler, 1974; Knutson and DeLuca, 1974; Rosol et al., 2000). Therefore, 1α-hydroxylase is upregulated during periods of hypocalcemia (Deluca, 1980).

Hypocalcemia also triggers the synthesis and release of PTH, a hormone produced by the chief cells of the parathyroid glands in response to signals by calcium-sensing receptors (Brown, 1991; Yarden et al., 2000) to regulate calcium within the bone, kidney, and small intestine (Laverty and Clark, 1989; Nys, 1993; Dacke et al., 2015). Specifically, PTH binds to its receptor on the cell surface of osteoblasts to alter their morphology and inactivate osteogenesis (Hurwitz, 1989). Additionally, PTH stimulation promotes calcium reabsorption in the kidney (Laverty and Clark, 1989). Simultaneously, PTH promotes the resorption of bone by stimulating osteoclasts to develop a ruffled border secreting enzymes and acid (Miller et al., 1984; Sugiyama and Kusuhara, 1994). If the hen is instead in a period of hypercalcemia, calcitonin is produced and secreted by the ultimobranchial glands (reviewed by: Dacke, 1979), acting in opposition of PTH. Calcitonin supports the development of bone by promoting osteoblasts to return to their original state, while inhibiting osteoclastic activity (Sugiyama and Kusuhara, 1993, 1996). As hens are under intensive calcium demands during a laying cycle, this hormone will primarily be responsible for downregulating the activity of PTH to support normokalemia. Calcium homeostasis is maintained throughout the life of the bird, from early embryogenesis to the end of a laying cycle. However, the production of E_2_ during sexual maturation promotes adaptive changes to calcium homeostasis to further support skeletal maintenance while stimulating eggshell formation.

### Development of the Skeletal Frame

Since the development of the skeletal frame begins during embryogenesis, the fully formed yolk also contains all the required Vitamin D_3_ and phosphorous *in ovo*, along with 1% of the total calcium required (Klassing, 1998a; 1998b). This is sufficient for the initial formation of hyaline cartilage, which occurs during the first 7–10 days of incubation (Richards and Packard, 1996). Once this source of calcium has been depleted, the chorioallantoic membrane (CAM) will mobilize calcium carbonate from the shell membrane. Meanwhile, carbonic anhydrase dissolves calcium from the shell, which diffuses through the shell membrane, binding to calcium-binding proteins on the surface of the ectoderm by E13 (Tuan and Scott, 1977). Thus, the role of E_2_ in breeder hens during eggshell formation directly impacts the success of skeletal development during embryogenesis, preventing metabolic disorders that occur later in the laying cycle of the offspring.

Once hatched, bones continue to grow in width and length via intermembranous and endochondral ossification, respectively (Pechak et al., 1986a, 1986b; Whitehead, 2004). During this period of growth, the structural bone develops as two types of lamellar bone: cortical and trabecular (Whitehead and Fleming, 2000). Intramembranous ossification contributes to bone thickness, with mesenchymal stem cells differentiating into bone until the period of sexual maturation (Pechak et al., 1986a, 1986b; Whitehead, 2004). Longitudinal growth occurs at the epiphyseal growth plates, with bone formation initiated in the lower hypertrophic zone. Chondroclasts reabsorb the cartilage matrix in this region to create space and support mineralization via the formation of hydroxyapatite crystals and infiltration of bone-building cells, referred to as osteoblasts (Whitehead, 2004). Bone development and remodelling progresses through the coordination of calcium deposition by osteoblasts and calcium breakdown triggered by osteoclasts (Roodman, 1999; Suda et al., 2001; Dacke et al., 2015). Prior to sexual maturation, the skeletal frame should have reached its maximum length and optimal thickness. This emphasizes the importance of managing the pullet prior to the increase in sex steroids to prevent the development of metabolic disorders later in the cycle, such as osteoporosis and cage layer fatigue, resulting in fragile skeletal structures (Whitehead and Fleming, 2000).

### Medullary Bone and the Laying Cycle

As mentioned above, the increase in circulating E_2_ associated with maturation leads to alterations in the control of calcium, Vitamin D, and PTH levels. The direct impact of E_2_ extends to the upregulation of 1,25(OH)_2_D_3_ receptors within the gut mucosa to improve the absorption of dietary sources of calcium. E_2_ has also been reported to increase the responsiveness of the kidney to PTH and promote the production of medullary bone (Pfeiffer and Gardner, 1938).

Particularly, upon activation of the reproductive axis, the hen terminates all further calcium deposition for the sole purpose of structural development. At this time, hens entering lay transition to the formation of the medullary bone (Bloom et al., 1941; Miller, 1992; Turner et al., 1994). This is a readily mobilizable source of calcium stored on the inner endosteal surface of the marrow cavities of long bones to provide calcium for future eggshell formation (Bloom et al., 1958; Mccoy and Reilly, 1996). This occurs primarily in the long bones, such as the femur and tibia but is also present in the pubic bone, ribs, ulna, toes, and scapula (Jacob and Pescatore, 2013). Studies have suggested medullary bone forms at the expense of structural bone since it has not been linked to direct improvements in strength (Whitehead and Wilson, 1992). However, additional findings have indicated a decline in fracture rate related to increased total content in the cavity (Fleming et al., 1998; Whitehead and Fleming, 2000), implying that medullary bone may play a beneficial role in the bone health of hens.

Medullary bone has been established to develop as early as 12–14 days preceding the first egg (Bloom et al., 1941; Miller, 1992; Turner et al., 1994), which is concomitant with the rise in E_2_ produced by the ovary during early follicular development (Benoit and Clavert, 1945; Common et al., 1948; Simkiss, 1961; Senior, 1974; Tanabe et al., 1981; Johnson et al., 1986; Miller, 1992). However, a recent study has demonstrated that the rise in E_2_ occurs at the time of age of first egg (AFE) in current commercial laying hens (Hanlon et al., 2021). Interestingly, this is further altered in broiler breeder hens, with the E_2_ peak occurring 2 weeks after the AFE (Takeshima, 2018; Takeshima et al., 2019). Thus, rather than require the peak concentration of E_2_, we hypothesize that there may be a threshold E_2_ concentration that must be achieved to trigger the initiation of medullary bone formation.

**FIGURE 4:**
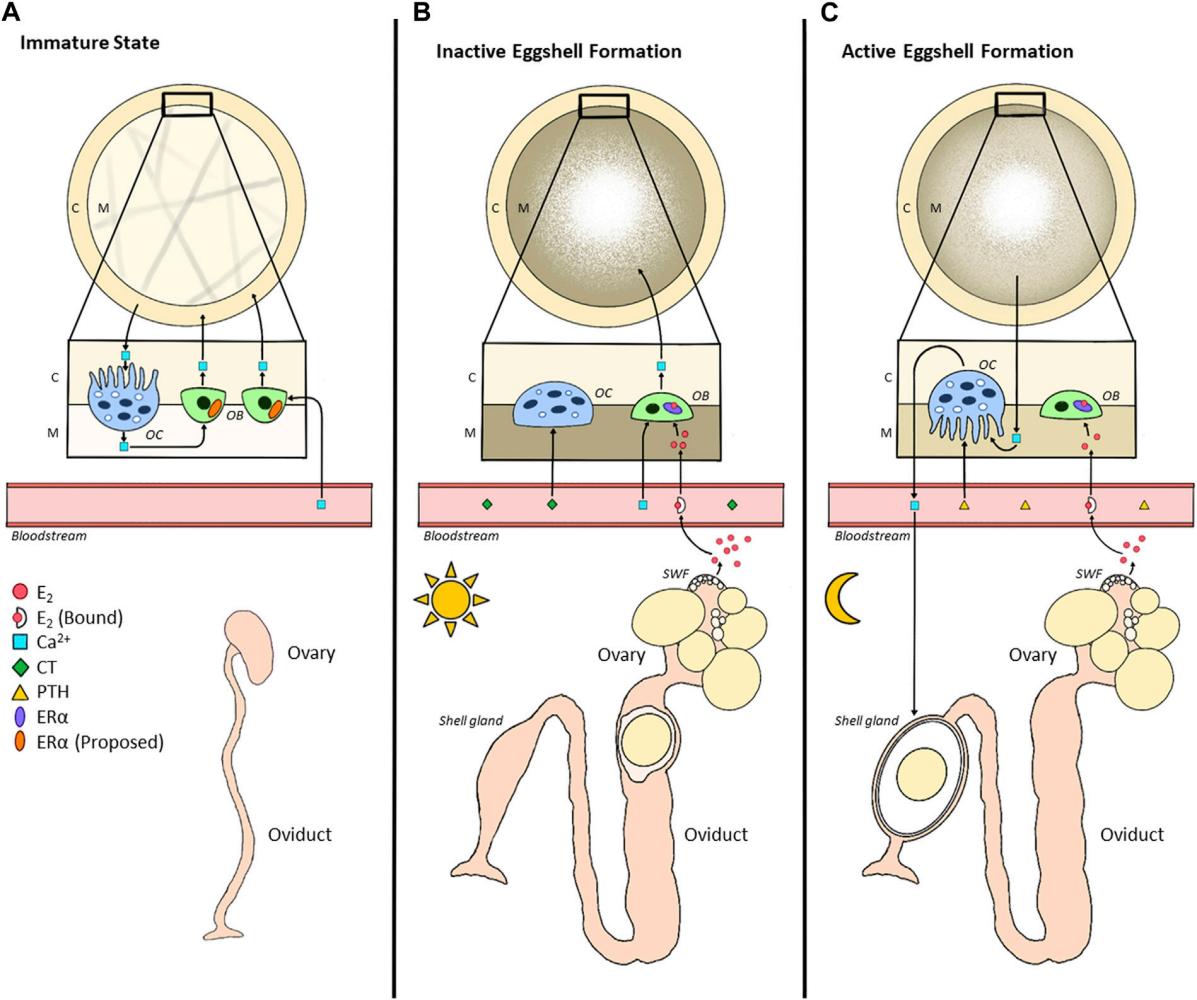
Alterations in Bone Metabolism between Immature and Mature States of Hens. **(A)**. During the immature state (pullet), calcium (Ca^2+^) is deposited by osteoblasts as cortical bone (C), serving as a structural source. Continuous bone remodelling occurs due to the resorptive capacity of osteoclasts. While the immature ovary produces very little E_2_ during this time, we propose that osteoblasts already express ERα in the immature state. Once the hen reaches maturation **(B,C)**, in each 24-h egg formation cycle, there will be inactive and active eggshell formation. **(B)**. Inactive eggshell formation occurs when the egg is present in the early portion of the oviduct (infundibulum, magnum, isthmus), usually during photophase. During this time, E_2_ enters the circulation where it binds to its carrier protein; a portion of free E_2_ then enters the cavity of the bone to bind to ERα expressed in osteoblasts. This triggers a morphological alteration in the shape of the osteoblast, interrupting the formation of cortical bone, instead opting for the formation of medullary bone (M), a readily mobilizable storage site of calcium for eggshell development. Calcium homeostasis is regulated by calcitonin (CT) at this time, downregulating the activity of osteoblasts via morphological alterations. **(C)**. Active eggshell formation begins once the egg enters the shell gland (uterus), typically during scotophase. Therefore, due to the unavailability of dietary calcium, medullary bone undergoes resorption to provide the shell gland with calcium. The morphological alterations in osteoclasts are triggered by the upregulation of parathyroid hormone (PTH). Simultaneously, although E_2_ binds to its receptor, the absence of additional calcium prevents the active formation of medullary bone at this time.

As ERα mRNA is expressed in osteoblasts, binding to E_2_ alters the morphology of these spindle-shaped cells and terminates the formation of structural bone while stimulating the initiation of medullary bone (Dacke et al., 1993, 2015). Simultaneously, osteoclasts are deactivated as they take on a cuboidal shape (Dacke et al., 2015). While PTH and 1,25(OH)_2_D_3_ levels typically regulate osteoclasts, E_2_ has been shown to indirectly influence the formation of this cell type (Oursler et al., 1991; Dacke et al., 1993). In mice models, E_2_ regulates the formation of osteoclasts by inhibiting a protein kinase pathway, resulting in indirect inhibition of the PTH-stimulated formation (Kanatani et al., 1998). However, as E_2_ also prevents apoptosis of osteocytes, this hormone supports the balance between the formation and resorption of bone during maturation (Tomkinson et al., 1998). Male quail treated with anti-estrogen displayed an elevation in the osteoclast population (Ohashi et al., 1987), suggesting that an inverse relationship between E_2_ and osteoclast activity is conserved in poultry. Unlike osteoblasts, early studies determining the presence of ERs on the surface of osteoclasts led to conflicting reports in the literature (Ohashi et al., 1991; Oursler et al., 1991). However, it is now largely accepted that osteoclasts will not express either ER subtype (Ohashi et al., 1991). Presently, most evidence suggests that E_2_ will play an indirect inhibitory role in bone resorption and a direct stimulatory role in bone formation (Oursler et al., 1991, 1993; Ali, 1992; Price and Russell, 1992; Figure 4).

In commercial laying hens, 2.2-g of calcium, or 10% of total calcium stores, is required for each eggshell (Nys et al., 1999; Bouvarel et al., 2011; Bain et al., 2016). While approximately 50% of this requirement can be covered by dietary calcium absorbed within the intestine daily (Kerschnitzki et al., 2014), about 33% is directly provided by the medullary bone (Bouvarel et al., 2011). Interestingly, this bone source stores ∼12% of the total bone calcium content within the hen’s body (Nys and le Roy, 2018). Since laying consists of active and inactive eggshell formation periods (Miller, 1977), medullary bone provides a source of calcium for the active shell formation, which primarily occurs during the scotophase (Clunies and Leeson, 1995). Specifically, the ovum reaches the shell gland ∼5 h post-ovulation, with eggshell formation occurring between 7 and 20 h post-ovulation (van de Velde et al., 1984).

Osteocalcin (OC) is a major non-collagenous protein produced by osteoblasts (Ugar and Karaca, 1999) and used to evaluate bone remodelling (Christenson, 1997; Ugar and Karaca, 1999; Seibel, 2005). Studies have suggested that calcium in the bone matrix is bound by OC (Christenson, 1997). However, comparisons of brown and white laying strains unexpectedly showed that brown strains have higher OC levels while white strains have higher E_2_ levels (Habig et al., 2021). This suggests that brown strains would have improved bone quality, despite the E_2_ differences observed. This hypothesis was confirmed by several studies (Eusemann et al., 2018a; Jansen et al., 2020; Habig et al., 2021). Thus, elevated OC in brown strains (Habig et al., 2021) may explain the lower prevalence of keel bone deformities (Eusemann et al., 2018a). This has led to speculated strain differences regarding the proportion of calcium mobilized from each source to form the eggshell. Habig et al. (2021) suggested that brown laying strains mobilize a more significant proportion of calcium from the bone compared to white layers. In contrast, white strains were hypothesized to meet calcium requirements via intestinal absorption. This may partially explain the improved feed conversion rate in white versus brown strains (Jansen et al., 2020). In addition, while the cumulative production was not found to differ between strains, the total proportion of eggshell was higher in white strains (Jansen et al., 2020), indicating that these hens may require more calcium for eggshell formation. Interestingly, while E_2_ did not differ between strains during lay, prior to maturation at 17 weeks of age (woa), white strains were found to have significantly higher E_2_ concentrations than the brown (Habig et al., 2021), possibly triggering a larger recruitment of calcium. This suggests that further studies are required to identify if there are differences in E_2_ involvement during maturation and active lay between white and brown strains.

### Calcium Homeostasis in the Aging Hen

Throughout the laying cycle, hens have been reported to undergo a progressive loss of mineralized structural bone (Webster, 2004). Previously, this was associated with declining E_2_ as the hen ages, causing medullary bone accumulation to cease and a breakdown of the cortical bone to supply calcium for the eggshell (Yamada et al., 2021). In fact, this decrease in E_2_ production coincides with the depletion of SWFs from the follicular pool, the associated decline in calcium availability for the shell formation, and slower follicular development, ultimately leading to the termination of lay. The depletion of medullary and cortical bone during the end of lay led to concerns regarding hen health and welfare, along with diminishing egg quality for the breeder and table egg industries. However, intensive genetic selection for extended laying periods resulted in commercial layer breeds adapted to meet these demands without impacting bone or eggshell quality (Hanlon et al., 2021, 2022). In fact, our latest studies suggest that modern strains of layers display recurrent elevations in E_2_ throughout a laying cycle. We hypothesize that this results in continuous stimulation of medullary bone formation, providing the hen with sufficient calcium storage to keep up with the demands of producing over 500 eggs in 100 weeks of life (Hanlon et al., 2021, 2022). Furthermore, these recurrent elevations in E_2_ were positively correlated with medullary bone mineral density (BMD) and negatively correlated with cortical BMD, yet cortical bone thickness did not change between 12 and 100 woa. Meanwhile, eggshell thickness and breaking strength did decline over time but never below an acceptable grade A quality (Hanlon et al., 2022). This is partially attributed to the selection for maintained egg size during extended lay (Bouvarel et al., 2011), since it has been determined that a constant quantity of calcium will be supplied to the shell regardless of the weight of the egg (Roland, 1979; Buss, 1988). Thus, understanding the impact of these additional E_2_ elevations will help understand the physiological needs associated with extended production cycles and adjust dietary calcium and Vitamin D accordingly to promote calcium uptake as the hen ages. As a matter of fact, current knowledge and guidelines, including NRC recommendations (NRC, 1994), may be outdated as it is based on reports that 1α-hydroxylase and intestinal calbindin production will decline with age contributing to the decline in Vitamin D metabolism (Abe et al., 1982; Joyner et al., 1987). However, as E_2_ promotes the uptake of 1,25(OH)_2_D_3_ within the digestive tract (Pfeiffer and Gardner, 1938), we propose that the unintended selection for recurrent E_2_ peaks has likely resulted in better Vitamin D absorption and synthesis.

### Calcium Tetany and Estradiol

Calcium tetany is a condition during which muscle weakness or even paralysis results from hypocalcemia (Turner and Debeer, 2009). While this is a common issue in commercial broiler breeder production systems, this disorder has been poorly characterized within the literature. It has been established that calcium tetany has similar outcomes to the well-described cage layer fatigue in layers, yet the timing of these conditions is vastly different. In the case of laying hens, this is an osteoporotic onset occurring at the end of the reproductive cycle and is typically associated with aging (Whitehead and Fleming, 2000). However, in broiler breeders, hens have been reported to exhibit these symptoms during sexual maturation up until the peak of lay, with a consequential spike in mortality observed (Turner and Debeer, 2009). Numerous management strategies have been recommended ranging from maximizing pullet uniformity to avoiding high calcium diets during the maturation period of breeders. This opposes the dietary recommendation in the laying hen industry, as a pre-lay diet is typically provided to these hens to slowly elevate calcium for the initial formation of the medullary bone ∼10 days prior to the onset of lay. This pre-lay diet includes ∼2% calcium, increasing to ∼4% once hens reach 5% production and remaining constant through the active laying phase (Lohmann-Tierzucht, 2015). Interestingly, breeding companies like Aviagen have suggested that broiler breeder hens should not be provided with elevated dietary calcium until they reach ∼5% production (Turner and Debeer, 2009).

The current literature has alluded that calcium regulation mechanisms initiated during sexual maturation are not responsive to higher calcium inclusion until the onset of lay has commenced (Turner and Debeer, 2009). In support of this hypothesis, studies in our laboratory identified that the initial E_2_ peak in the laying hen occurs at the time of AFE (Hanlon et al., 2021). In contrast, this elevation in E_2_ typically associated with sexual maturation occurred 2 weeks after the AFE in the broiler breeder hen (Takeshima, 2018; Takeshima et al., 2019). This could potentially explain the differing responsiveness to dietary calcium between laying hens and broiler breeders. As eggshell formation is initiated, calcium levels are depleted, resulting in a decline in PTH levels. This significantly reduces calcium excretion and increases 1,25(OH)_2_D_3_ levels to stimulate calcium absorption (Pfeiffer and Gardner, 1938; Laverty and Clark, 1989). However, without an early elevation in E_2_ to enhance 1,25(OH)_2_D_3_ and calbindin receptors in the intestine, along with the morphological alterations to osteoblast and osteoclast activity, providing dietary calcium too early in broiler breeder hens will result in extreme calcium imbalances. Without the influences of E_2_, calcium retention will not be able to compensate for the excretion rate. However, further studies are required to validate this hypothesis and understand the integration between E_2_, AFE, and calcium supplementation to provide better management recommendations in broiler breeder hens.

### Osteoporosis and Estradiol

Bone fractures and keel bone deviations have long been attributed to high rates of lay (Gregory and Wilkins, 1989; Rufener and Makagon, 2020), although the etiology of bone disorders has recently been an area of discussion (Jansen et al., 2020; Dunn et al., 2021). However, it is undisputed that keel bone fractures can occur in up to 97% of commercial laying flocks (Wilkins et al., 2011; Richards et al., 2012; Petrik et al., 2014; Heerkens et al., 2016). In laying hens, osteoporosis was previously associated with caged housing systems, hence its designation as cage layer fatigue (Grumbles, 1959). However, the onset of osteoporosis and declining bone quality is much more complex than simply being under the control of the environment. E_2_ has been negatively associated with cortical BMD while positively correlated with medullary BMD (Hanlon et al., 2022), indicating that the onset of lay predisposes hens to the onset of osteoporosis. This is supported by evidence suggesting that medullary bone adds no structural integrity (Wilson and Thorp, 1998). Regardless, declining E_2_ in the aging hen results in decreased calcium absorptive capacity, which leads to an overall negative association between E_2_ and osteoporosis (Abe et al., 1982; Joyner et al., 1987). Altogether, these reports suggest the relationship between this hormone and metabolic condition may be much more complex. The sustained release of the GnRH agonist, deslorelin acetate, diminishes the prevalence of keel bone damage at the expense of the laying cycle (Eusemann et al., 2018b). This supports the hypothesis that lower E_2_ levels are critical for maintaining skeletal structure integrity (Beck and Hansen, 2004). However, this comes at the expense of egg production and is thus impractical for commercial production. Although research from the early 1960s suggested that bone fragility was induced by E_2_-treatment, resulting in a thinner cortex (Urist and Deutsch, 1960), recent studies indicate that this may no longer be a primary concern (Hanlon et al., 2022).

Interestingly, osteoporosis has also been associated with obesity (Rosen and Bouxsein, 2006; Zhao et al., 2007). Specifically, visceral fat has been shown to play a detrimental role in the structural integrity of bone (Janicka et al., 2007; Gilsanz et al., 2009). This is consistent with the increased risk of developing osteoporosis under FLHS (Pardee et al., 2012; Yilmaz, 2012). Inducing FLHS via HELP diets results in upregulated bone turnover and detectable damage to skeletal integrity (Jiang et al., 2013). This suggests an additional link between the roles of E_2_ on fat metabolism and bone development. As a matter of fact, the inclusion of long-term dietary lipids has been detrimental to bone remodelling (Liu et al., 2003, 2004), bone formation rate (Watkins et al., 1997), and bone mineral content (Liu et al., 2004). Specifically, OC alleviated liver damage caused by this high-lipid diet (Wu et al., 2021). OC levels are highest in the pullet phase, slowly declining to reach the lowest levels at the end of a laying cycle (Jiang et al., 2013; Nys and le Roy, 2018), thus displaying an inverse relationship with E_2_ during maturation. Wu et al. (2021) proposed that OC can counteract the onset of FLHS in older laying hens. Taking that one step further, we hypothesize that the recurrent elevations in E_2_ we reported (Hanlon et al., 2021) are key to sustaining extended reproduction and balancing the activation of bone and liver metabolism to support egg formation.

## Conclusion

In conclusion, the involvement of E_2_ in activating the reproductive axis, liver metabolism, and medullary bone formation demonstrates that this hormone contributes to and may coordinate all aspects of egg formation. However, while the success of the breeder and table egg industries is highly dependent on E_2_, little research has considered the underlying endocrinological alterations responsible for improvements in laying rate. These effects are primarily mediated through ERα, resulting in the production of yolk proteins in the liver and the storage of calcium in the medullary bone while also coordinating their transport to the reproductive tract. However, by diverting nutrients and energy towards egg formation, E_2_ can also contribute to the onset of metabolic disorders, such as FLHS and calcium tetany. Nonetheless, decades of genetic selection for egg production have impacted the patterns of E_2_ production, which may have helped accommodate the further demands in modern strains of commercial laying hens. Interestingly, this may not be the case in broiler breeder hens undergoing selection for rapid growth, as a delay in the initial rise in E_2_ is associated with calcium tetany during sexual maturation. Thus, it is apparent that rather than absolute concentrations, the timing of E_2_ production is key to successful reproduction, which requires tight coordination with metabolic processes to prevent liver and skeletal damage. As ERs are present in several additional metabolic organs, including adipose and kidney in mammals, future investigations should consider the impact this hormone may have on the control of energy partitioning between growth and reproductive process, particularly during the preparation for lay.
